# Short-Term Effects of Particulate Matter on Stroke Attack: Meta-Regression and Meta-Analyses

**DOI:** 10.1371/journal.pone.0095682

**Published:** 2014-05-06

**Authors:** Xiao-Bo Yu, Jun-Wei Su, Xiu-Yang Li, Gao Chen

**Affiliations:** 1 Department of Neurosurgery, the Second Affiliated Hospital of Zhejiang University School of Medicine, Hangzhou, P.R. China; 2 Key Laboratory of Infectious Diseases Ministry of Public Health of China, the First Affiliated Hospital of Zhejiang University School of Medicine, Hangzhou, P.R. China; 3 Department of Public Health, Zhejiang University, Hangzhou, P.R. China; Tsinghua University, China

## Abstract

**Background and Purpose:**

Currently there are more and more studies on the association between short-term effects of exposure to particulate matter (PM) and the morbidity of stroke attack, but few have focused on stroke subtypes. The objective of this study is to assess the relationship between PM and stroke subtypes attack, which is uncertain now.

**Methods:**

Meta-analyses, meta-regression and subgroup analyses were conducted to investigate the association between short-term effects of exposure to PM and the morbidity of different stroke subtypes from a number of epidemiologic studies (from 1997 to 2012).

**Results:**

Nineteen articles were identified. Odds ratio (OR) of stroke attack associated with particular matter (“thoracic particles” [PM_10_]<10 µm in aerodynamic diameter, “fine particles” [PM_2.5_]<2.5 µm in aerodynamic diameter) increment of 10 µg/m^3^ was as effect size. PM_10_ exposure was related to an increase in risk of stroke attack (OR per 10 µg/m^3^ = 1.004, 95%CI: 1.001∼1.008) and PM_2.5_ exposure was not significantly associated with stroke attack (OR per 10 µg/m^3^ = 0.999, 95%CI: 0.994∼1.003). But when focused on stroke subtypes, PM_2.5_ (OR per 10 µg/m^3^ = 1.025; 95%CI, 1.001∼1.049) and PM_10_ (OR per 10 µg/m^3^ = 1.013; 95%CI, 1.001∼1.025) exposure were statistically significantly associated with an increased risk of ischemic stroke attack, while PM_2.5_ (all the studies showed no significant association) and PM_10_ (OR per 10 µg/m^3^ = 1.007; 95%CI, 0.992∼1.022) exposure were not associated with an increased risk of hemorrhagic stroke attack. Meta-regression found study design and area were two effective covariates.

**Conclusion:**

PM_2.5_ and PM_10_ had different effects on different stroke subtypes. In the future, it's worthwhile to study the effects of PM to ischemic stroke and hemorrhagic stroke, respectively.

## Introduction

Many studies regarded air pollution exposure as an important factor of hospitalization and mortality worldwide. PM, playing an important role in pollutants of major public health concern, had been confirmed that it could impair the respiratory and cardiovascular system through a series of changes in autonomic nervous system activity [1] and systemic inflammation [2], giving rise to alterations in oxidative stress [3], [4], hematologic activation [5] and vascular endothelial dysfunction [6]. Most researches regarded PM_10_ and PM_2.5_ as major harmful PMs.

Nevertheless, short-term effects of PM exposure on cerebral vessels were uncertain. Wordley et al. [7] and Tsai et al. [8] found that PM_10_ was associated with daily stroke attack positively. While, in the works of Chan et al. [9], Henrotin et al. [10] and Andersen et al. [11], no significant association was demonstrated between PM_10_ and hemorrhagic stroke attack. Similarly, analyses on the relationship between PM_2.5_ and stroke attack also appeared to divergent results. Villeneuve et al. [12] found PM_2.5_ exposure wasn't related to an increased risk of ischemic stroke attack (OR per 10 µg/m^3^ = 1.052, 95%CI: 0.996∼1.160), while Wellenius et al. [13] found a positive association between PM_2.5_ exposure and ischemic stroke attack (OR per 10 µg/m^3^ = 1.278, 95%CI: 1.079∼1.525).

Our previous research focused on the association between PM exposure and stroke attack in two study designs (time-series design and case-crossover design), and the result indicated that the effects of PM to stroke attack varied in different study designs [14]. However, in addition to study design, there were still many other covariates (e.g. age, gender, economic condition, area, lags times, historical disease and temperature) among studies, which could influence the results. Of special interest was that whether PM can act differently on different stroke subtypes. So in this article we determined to do meta-analyses, meta-regression and subgroup analyses of association between PM and different stroke attack.

## Methods

### 1. Studies selection

We identified studies published in English and Chinese up to March 2013, by literature search using PubMed, Web of Science, MEDLINE, Google Scholar, China National Knowledge Infrastructure (CNKI) and reference lists of relevant articles. Search terms included “Air Pollution/Particulate Matter” plus “Cardiovascular disease/Stroke”, besides, key terms “hospitalization/Hospital Administration/Emergencies/Morbidity”, “cardiovascular disease” were used to enlarge the searching range. We chose ICD9: 430–438 or ICD10: I60–I69 as the definition of “stroke” or “cerebrovascular disorders”, ICD9: 430–432 or ICD10: I60–I62 for “hemorrhagic stroke”, ICD9: 433–434 or ICD10: I63–I66 for “ischemic stroke”.

Eligible studies were selected by two reviewers (X.L., J.S.) independently according to following inclusion criteria: (1) The outcome focused on the effect of PM to stroke or cerebrovascular disease (2) Published full-text articles (3) focused on PM_10_ and/or PM_2.5_ (4) Studies with similar effects [e.g. risk ratios (RR), 95% CIs] that could approximate ORs. The exclusion criteria were: (1) Duplications (2) Reviews or Meta-analysis (3) Long-term effects articles (4) air pollution from industrial and occupational environment (5) Articles that did not provide calculable or reported ORs and 95% CIs. The two reviewer reached consensus on the eligibility of each study. When there was disagreement, a third reviewer (G.C.) took the final decision.

Using a standardized form, data from eligible studies were extracted by two reviewers (X.L., J.S.) independently, From each study, we collected name of first author, year of publication, number of participants, PM, country, age, gender, types of stroke, lags of air pollutants' concentrations, study design and ORs or RRs with 95% CI. In Meta-regression, covariates were gender, area, lag times, study design and research period. Gender was represented as female, male and the whole population. Areas included Asia, Europe, North America and Oceania. Lag times were the same day on stroke attack and the previous 1, 2, 3, 4, 5 day. Study designs were classified into time-series and case-crossover designs. Research period was divided into early stage (1992–1997), middle stage (1997–2004) and late stage (2004–2009). Given there were 5 covariates (gender, area, lag times of air pollutants' concentrations, study design and research period), we used Meta-regression model to detect any possible influence factors (*P*<0.05).

### 2. Statistical analysis

Pooled ORs of PM with 95%CI for stroke attack were calculated by using the fixed or random effects Meta-analysis of with Q and I^2^ statistics given as the chosen measure of heterogeneity (The null hypothesis of this test is homogeneity). The Q and I^2^ statistics were used to assess heterogeneity, where *P*≤0.05 or I^2^>50% were considered as significant heterogeneity. We presented random effects pooled estimates when heterogeneity was detected; otherwise, we used the fixed model. We also produced forest plots to show ORs from each of the individual studies included in the meta-analyses and the estimation of the pooled OR. The sizes of the markers of each OR in the plots represent the relative weight each study contributed to the pooled estimation. We assessed publication bias visually through funnel plots and a weighted Egger's test. We also performed sensitivity analyses, whereby each article was omitted in turn, recalculating the pooled estimates under extreme conditions. Moreover, Meta-regression analysis was performed to figure out whether the association between PM and stroke attack was influenced by covariates. With a positive Meta-regression coefficient presented (*P*≤0.05), we could recognize the influence of the given factor. All analyses were performed using software STATA version 12.0 (StataCorp LP, College Station, TX, USA).

## Results

### 1. Study selection and data extraction

**Figure 1:**
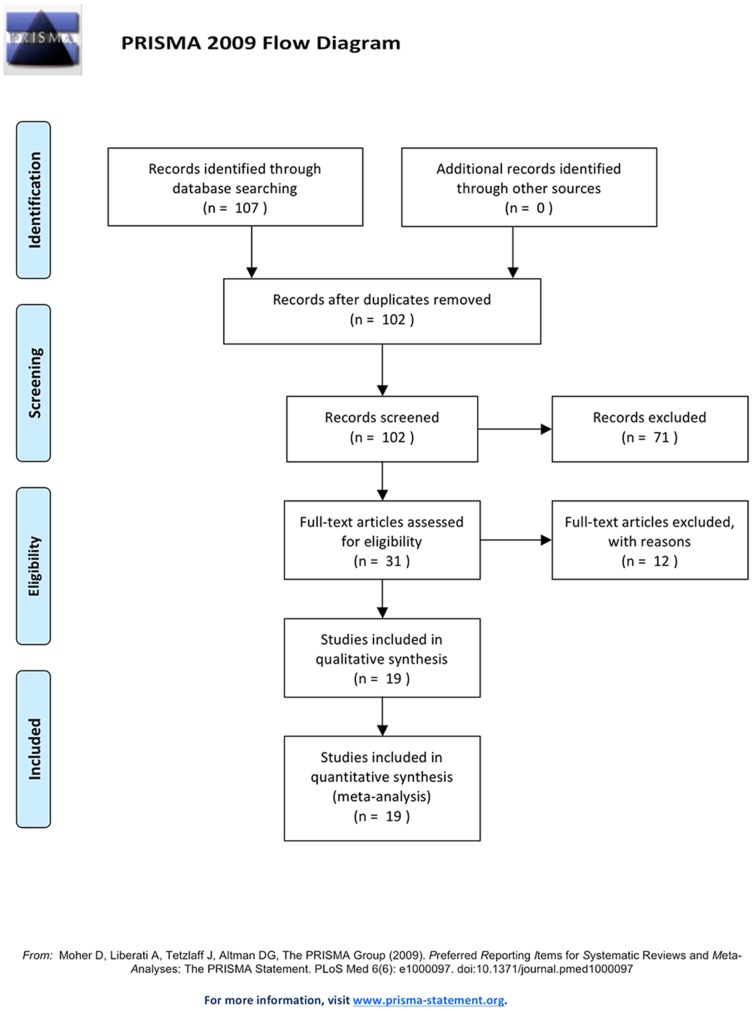
Flow chart of the selection process.

A total of 107 potentially relevant researches were identified by searching electronic databases and reference lists. 19 full-text articles were eligible for inclusion in this analysis and data were extracted from these studies [7]–[13], [15]–[26]. The details of the selection process were presented in a flow chart in Figure 1. Publication years ranged from 1997 and 2012. In total, 9 countries were involved including Australia, Canada, China, Denmark, Finland, France, Italy, UK and US. 14 articles focused on PM_10_ and 9 were about PM_2.5_. In our previous research [14], we only extracted adjusted maximum effective value in each study. However, in this article, we took advantage of all the effect values (33 for PM_2.5_, 68 for PM_10_, 29 for hemorrhagic stroke, 42 for ischemic stroke) that fulfilled our study aim. The basic overview of the 19 included articles is given in Table 1.

**Table 1:** The basic overview of the 19 included articles.

First author(year of publication)	Particulate matter	Case number	Country	Age	Gender	Study Design	Lag*	Research period	Types of stroke
Wordley(1997) [7]	PM_10_	NM	UK	all	all	time-series	0	1992–1994	stroke
Wong(1999) [15]	PM_10_	NM	China	all	all	time-series	2	1994–1995	stroke
Linn(2000) [16]	PM_10_	108114	US	>29	all	time-series	0	1992–1995	stroke
Tsai(2003) [8]	PM_10_	16067	China	all	all	case-crossover	0	1997–2000	HS and IS
Chan(2006) [9]	PM_2.5_、 PM_10_	8582	China	all	all	time-series	0, 1, 2, 3	1997–2002	stroke, HS and IS
Dominici(2006) [17]	PM_2.5_	11500000	US	≥65	all	time-series	0	1999–2002	stroke
Jalaludin(2006) [18]	PM_2.5_、 PM_10_	20634	Australia	≥65	all	time-series	0, 1, 2, 3, 4	1997–2001	stroke
Villeneuve(2006) [19]	PM_2.5_、 PM_10_	12034	Canada	≥65	all	case-crossover	0, 1, 3	1992–2002	HS and IS
Henrotin(2007) [10]	PM_10_	1630	France	>40	F, M, all	case-crossover	0, 1, 2, 3	1994–2004	HS and IS
Bell(2008) [20]	PM_2.5_、 PM_10_	11466	China	all	all	time-series	0, 1, 2, 3, 0–3	1995–2002	stroke
Guo(2008) [21]	PM_10_	2990	China	all	all	case-crossover	0, 1, 2, 3	2004–2006	stroke
Lisabeth(2008) [22]	PM_2.5_	3508	US	≥45	all	time-series	0, 1	2001–2005	IS
Halonen(2009) [23]	PM_2.5_	10383	Finland	≥65	all	time-series	0, 1, 2, 3, 0–4	1998–2004	stroke
Ye(2009) [24]	PM_10_	699	China	all	all	case-crossover	0	2002–2004	HS
Andersen(2010) [11]	PM_10_	6369	Denmark	all	all	case-crossover	0, 1, 2, 3, 4, 0–4	2003–2006	HS and IS
Vidale(2010) [25]	PM_10_	759	Italy	all	all	time-series	0, 1, 2, 3, 4, 5	2000–2003	IS
Villeneuve(2012) [12]	PM_2.5_	5927	Canada	>18	all	case-crossover	0, 1, 3	2003–2009	stroke, HS and IS
Wellenius(2012) [13]	PM_2.5_	1705	US	≥21	all	case-crossover	0–1	1999–2008	IS
Willocks(2012) [26]	PM_10_	NM	UK	all	all	time-series	0, 1, 2, 3, 4, 5	2000–2006	stroke

NM indicates not mentioned; PM_10_, particular matter with aerodynamic diameter ≤10 µm; PM_2.5_, particular matter with aerodynamic diameter ≤2.5 µm; UK, the United Kingdom; US, the United States; F, female; M, male; HS, hemorrhagic stroke; IS, ischemic stroke.

*lag was the timing of the exposure; Lag0, the same day exposure; Lag1, exposure the day before; Lag2, exposure the 2 days before; Lag3, exposure the 3 days before; Lag4, exposure the 4 days before; Lag5, exposure the 5 days before; Lag0–3, mean exposure of previous 3 days and the same day; Lag0–4, mean exposure of previous 4 days and the same day.

### 2. Meta-analysis of different PMs exposure to different stroke types

#### 2.1 Effects of PM_2.5_、 PM_10_ exposure to stroke attack

**Figure 2:**
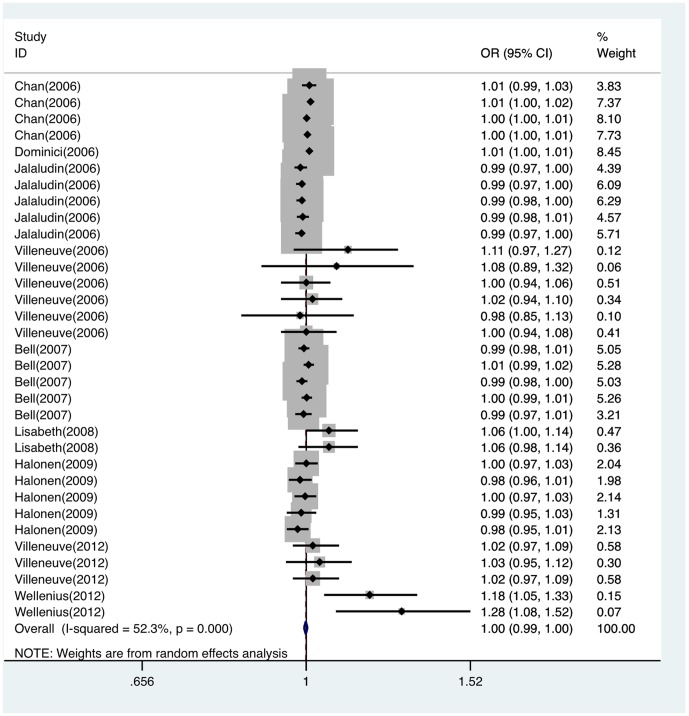
Forest plot of ORs for the association between PM_2.5_ and stroke attack. OR indicates odds ratio; CI, confidence interval.

**Figure 3:**
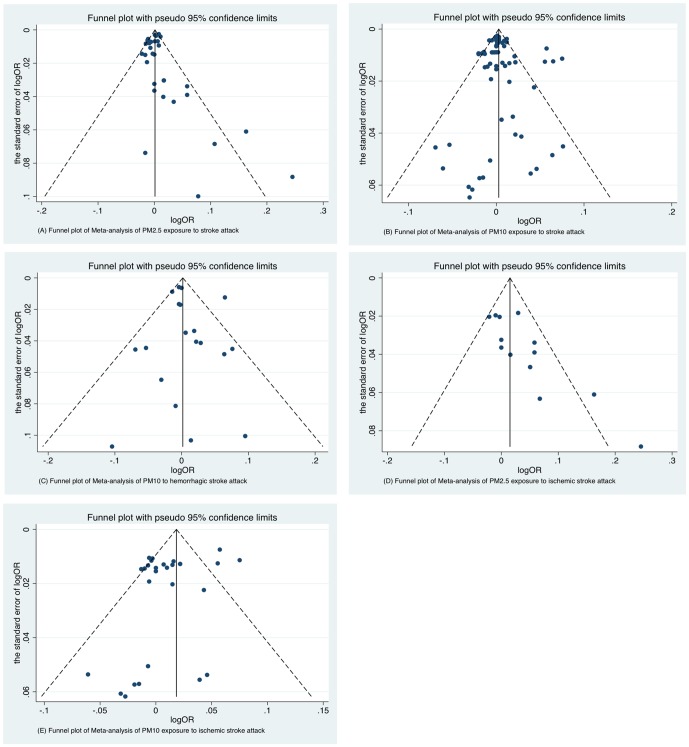
Funnel plots of Meta-analysis in different particular matters to different stroke types. OR indicates odds ratio. (A) Funnel plot of Meta-analysis of PM_2.5_ exposure to stroke attack. (B) Funnel plot of Meta-analysis of PM_10_ exposure to stroke attack. (C) Funnel plot of Meta-analysis of PM_10_ exposure to hemorrhagic stroke attack. (D) Funnel plot of Meta-analysis of PM_2.5_ exposure to ischemic stroke attack. (E) Funnel plot of Meta-analysis of PM_10_ exposure to ischemic stroke attack.

There were nine articles (containing 33 studies) that referred to the association of PM_2.5_ and stroke attack. Since heterogeneity existed among studies (Q = 67.09, *P*<0.05), the random effect model was conducted to calculate a pooled OR with 95%CI. The Meta-analysis results indicated that PM_2.5_ exposure wasn't related to an increased risk of stroke attack (OR per 10 µg/m^3^ = 0.999, 95%CI: 0.994∼1.003), Forest plot and Funnel plot were shown in Figure 2 and Figure 3(A), respectively. Egger's test (*t* = 0.98, *P* = 0.336) didn't find evidence of publication bias. Sensitivity analysis suggested that no individual study significantly affected the pooled effect size, indicating that the results for PM_2.5_ and daily stroke attack were statistically robust (Table S1 in File S1).

**Figure 4:**
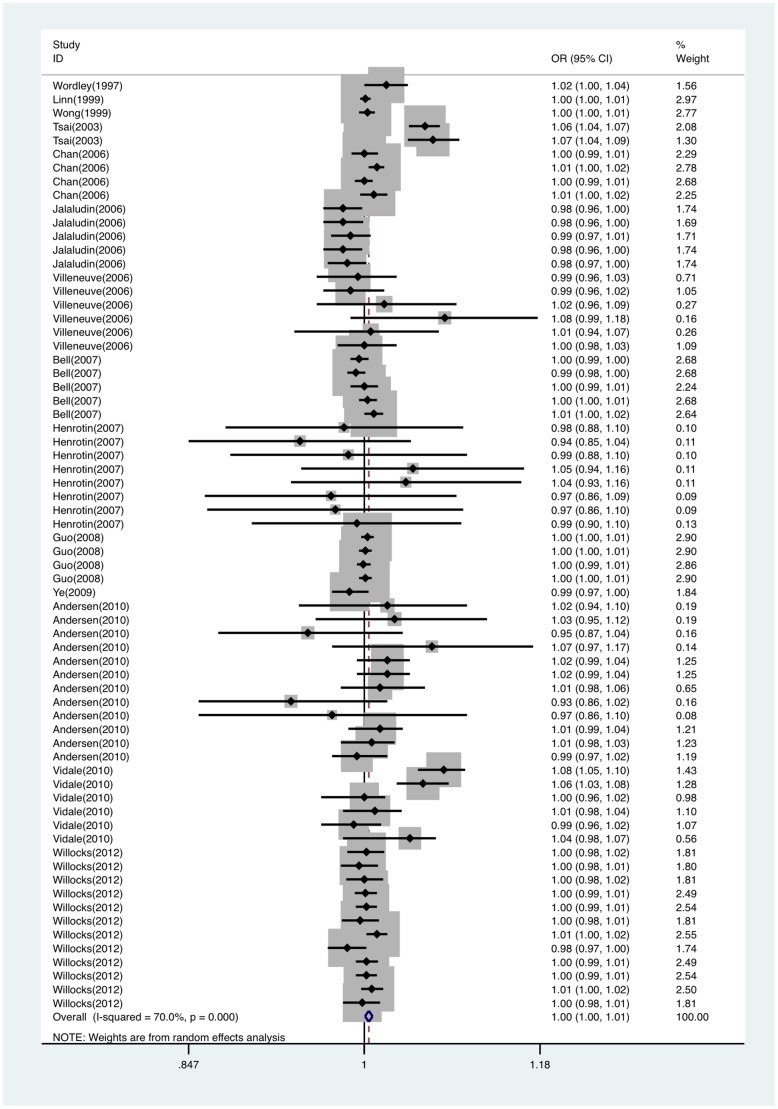
Forest plot of ORs for the association between PM_10_ and stroke attack. OR indicates odds ratio; CI, confidence interval.

Fourteen articles (containing 68 studies) were included. The heterogeneity was significant (Q = 223.25, *P*<0.05). With the random effect model, we pooled all 68 studies into the meta-analysis and found PM_10_ exposure was statistically significantly associated with an increased risk of stroke attack (OR per 10 µg/m^3^ = 1.004; 95%CI, 1.001∼1.008). Forest plot was shown in Figure 4. Funnel plot was shown in Figure 3(B). Egger's test supported that publication bias was unlikely (*t* = 0.80, *P* = 0.427). Sensitivity analysis showed that results for PM_10_ and stroke attack were not robust to the inclusion of Tsai study [8] and Vidale study [25] (Table S1 in File S1).

#### 2.2 Effects of PM_2.5_、 PM_10_ exposure to hemorrhagic stroke attack

Since the effect values of all studies included were not statistically significant, so it had no sense to do the Meta-analysis. As a result, we could not just simply determine the association between PM_2.5_ exposure and hemorrhagic stroke attack.

**Figure 5:**
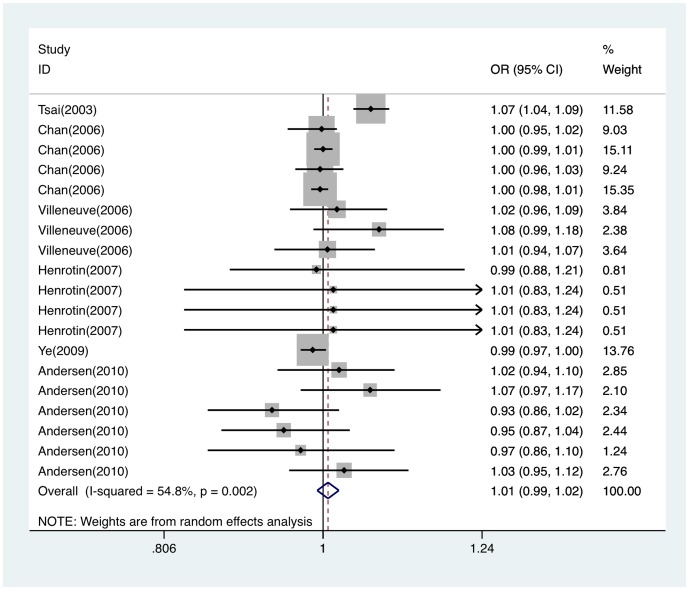
Forest plot of ORs for the association between PM_10_ to hemorrhagic stroke attack. OR indicates odds ratio; CI, confidence interval.

We conducted meta-analysis for nineteen combinative effects of PM_10_ and hemorrhagic stroke attack. Heterogeneity was detected (Q = 39.82, *P* = 0.002) through heterogeneity test. With random effect model, we found no evidence for the association between PM_10_ exposure and hemorrhagic stroke attack (OR per 10 µg/m^3^ = 1.007; 95%CI, 0.992∼1.022). Forest plot was shown in Figure 5, Funnel plot was shown in Figure 3(C). Egger's test showed that publication bias was unlikely in the meta-analysis on association between PM_10_ and hemorrhagic stroke attack (*t* = 0.71, *P* = 0.487). Sensitivity analysis suggested the results for PM_10_ and hemorrhagic stroke attack were statistically robust (Table S1 in File S1).

#### 2.3 Effects of PM_2.5_、 PM_10_ exposure to ischemic stroke attack

**Figure 6:**
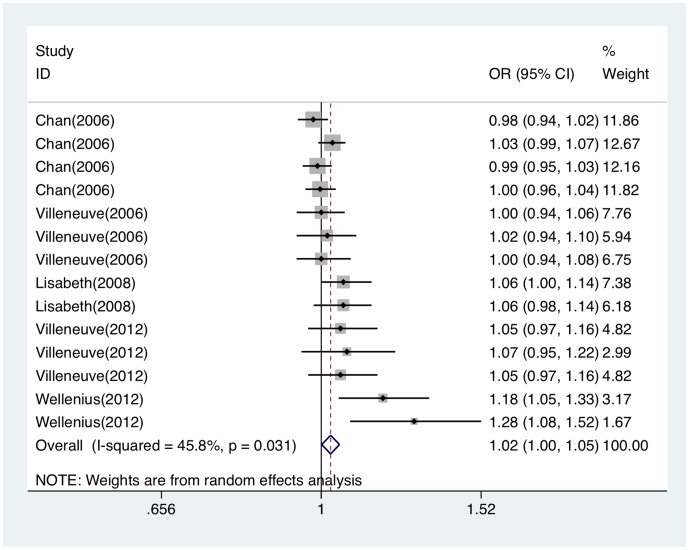
Forest plot of ORs for the association between PM_2.5_ and ischemic stroke attack. OR indicates odds ratio; CI, confidence interval.

Heterogeneity was observed among five articles (Q = 24.00, *P* = 0.031). With random effect model, PM_2.5_ exposure was related to the risk of ischemic stroke attack (OR per 10 µg/m^3^ = 1.025; 95%CI, 1.001∼1.049). Forest plot was shown in Figure 6. Funnel plot was shown in Figure 3(D). Egger's test (*t* = 3.71, *P* = 0.003) showed publication bias existed among studies. Sensitivity analysis showed that results for PM_2.5_ and ischemic stroke attack were not robust to the inclusion of Lisabeth study [22], Villeneuve study [12] and Wellenius study [13] (Table S1 in File S1).

**Figure 7:**
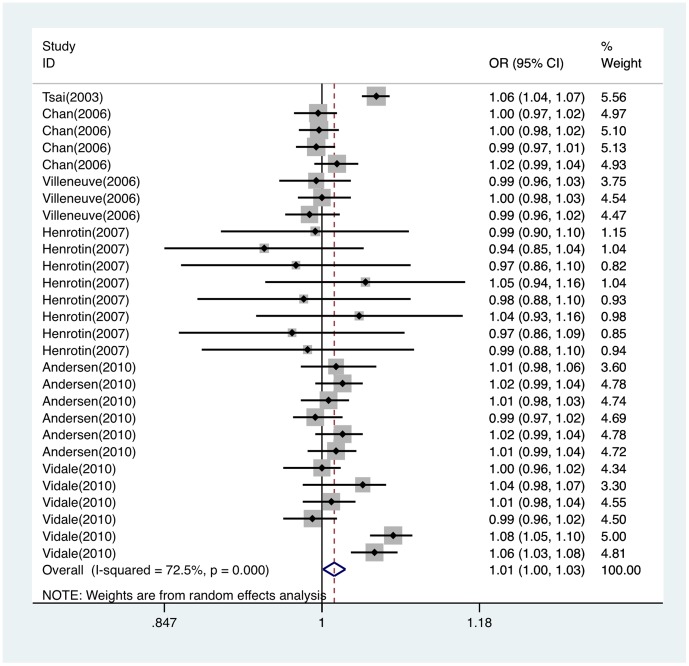
Forest plot of ORs for the association between PM_10_ and ischemic stroke attack. OR indicates odds ratio; CI, confidence interval.

Significant heterogeneity existed among six articles included (Q = 98.04, *P* = 0.000). Association was demonstrated between PM_10_ exposure and ischemic stroke attack (OR per 10 µg/m^3^ = 1.013; 95%CI, 1.001∼1.025) using random effect model. Forest plot was shown in Figure 7. Funnel plot was shown in Figure 3(E). Egger's test (*t* = −1.61, *P* = 0.120) indicated no publication bias existed among studies of association between PM_10_ and ischemic stroke attack. Sensitivity analysis showed that results for PM_10_ and ischemic stroke attack were not robust to the inclusion of Andersen study [11] and Vidale study [25] (Table S1 in File S1).

### 3. Results of Meta-regression of different PMs exposure to different stroke types

We had detected heterogeneity among studies about PM_2.5_ exposure to stroke attack, PM_10_ exposure to stroke attack, PM_10_ exposure to hemorrhagic stroke attack, PM_2.5_ exposure to ischemic stroke and PM_10_ exposure to ischemic stroke. Meta-regressions were done in these studies to detect the influence factors. (Table S2 in File S1).

When studying PM_2.5_ exposure to stroke attack, we found study design (coefficient 0.032, 95% CI 0.002 to 0.062, *P* = 0.035) the influence factor. Among studies of PM_10_ exposure to stroke attack, area (coefficient 0.007, 95% CI 0.002 to 0.012, *P* = 0.007) were found to be the possible influence factor. (Table S2 in File S1).

### 4. Subgroup analyses

Results above supported that design was the main covariate of studies about PM_2.5_ exposure to stroke attack, so we divided these studies into subgroups (time-series group and case-crossover group) according to study design. Case-crossover group revealed the positive association between PM_2.5_ exposure and stroke attack (OR per 10 µg/m^3^ = 1.029; 95%CI, 1.003∼1.055). And area was found to be the main covariate of studies about PM_10_ exposure to stroke attack, subgroups (Asia, Europe, North America and Oceania) analysis showed that in Asia and Europe, PM_10_ was associated with stroke attack (Table 2).

**Table 2:** Meta-analysis of subgroup studies.

	Covariate	Level	Number of articles	Number of studies	Model	Pooled OR (95%CI)
The effect of PM_2.5_ to stroke attack	study design	time-series study	6	22	random effect model	0.998 (0.993∼1.002)
		case-crossover study	3	11	fixed effect model	1.029 (1.003∼1.055)
The effect of PM_10_ to stroke attack	area	Asia	6	17	random effect model	1.006 (1.000∼1.011)
		Europe	5	39	random effect model	1.008 (1.002∼1.014)
		North America	2	7	fixed effect model	1.001 (0.996∼1.006)
		Oceania	1	5	fixed effect model	0.982 (0.974∼0.990)

PM_2.5_ indicates particular matter with aerodynamic diameter ≤2.5 µm; PM_10_ indicates particular matter with aerodynamic diameter ≤10 µm; OR, odds ratio; CI, confidence interval.

## Discussion

The pooled results of meta-analyses showed that PM_10_ exposure was related to an increase in risk of stroke attack (OR per 10 µg/m^3^ = 1.004, 95%CI: 1.001∼1.008) and PM_2.5_ exposure was not significantly associated with stroke attack (OR per 10 µg/m^3^ = 0.999, 95%CI: 0.994∼1.003). So it's meaningful to explore the effects of PM_10_ and PM_2.5_ to stroke attack, separately.

However, as we all know, ischemic stroke and hemorrhagic stroke are two different types of stroke. The mechanisms differ from each other. So the PM may affect different strokes in different ways. Thus, it is valuable to explore the association between PM and ischemic stroke or hemorrhagic stroke, respectively. This was the first Meta-analysis that focused on the association between particular matter and different stroke subtypes attack. Our research showed that PM_2.5_ (OR per 10 µg/m^3^ = 1.025; 95%CI, 1.001∼1.049) and PM_10_ (OR per 10 µg/m^3^ = 1.013; 95%CI, 1.001∼1.025) exposure were statistically significantly associated with an increased risk of ischemic stroke attack, while PM_2.5_ (all the studies showed no significant association) and PM_10_ (OR per 10 µg/m^3^ = 1.007; 95%CI, 0.992∼1.022) exposure were not associated with an increased risk of hemorrhagic stroke attack. We inferred that this phenomenon might be caused by the different formation mechanisms of different stroke types. For the biological mechanisms of PM to cerebrovascular disease are not clearly clarified at present, we can just supposed that PM's effect to ischemic stroke attack might be regulated by systemic inflammation and the activation of coagulation system, which leading to atherosclerosis, vasoconstriction, increase of fibrinogen and acceleration the formation of acute thrombus. And PM's effect to hemorrhagic stroke attack might be caused by systemic inflammation, giving rise to alterations in oxidative stress, vascular endothelial damage and rupture of plaque. According to our results, it might be easier for PM to activate coagulation system and constriction of blood vessels than simply destroy the vascular endothelial. But it was just a hypothesis that needed to be tested.

Heterogeneity in the studies we retrieved could come from inherent differences between study settings, as well as from differences in age, gender, area, lag times of air pollutants' concentrations, study design and so on. With respect to these influence factors, we used randomized effect model to minimize the heterogeneity and do Meta-regression to find the possible covariates. As a result, we only found design and area were the influence factors in the studies.

Sensitivity analysis revealed that results for PM and ischemic stroke were not robust, but results for PM and hemorrhagic were more robust than the former. Except for the studies of PM_2.5_ and ischemic stroke, all the studies showed a symmetric inverted funnel shape, which indicated publication bias was unlikely. Besides, Egger's test, of which the results support funnel shape, was used to quantitatively assess the publication bias.

In this research, the following issues needed to be considered: (1) there were limitations for selecting studies. First, Meta-analysis articles [27]–[30] were not included in our research, because the methods authors used were different from ours, and they only provided pooled effects of PM exposure to stroke attack. Second, long-term effects articles [31]–[33] were not included, for there existed more covariates [e.g. body-mass index (BMI), smoking status, blood pressure, educational level, household income and historical disease] in long-term effect articles. Third, articles [34]–[37] proving quantitative relationship between PM and stroke attack but didn't provide calculable or reported ORs and 95% CIs were removed, too. Hence, removing these three kinds of articles might decrease bias but could lose some evidence of the association between PM and stroke attack. (2) Measurement of air pollution exposure varies within and between studies. The air pollution monitor apparatus itself had measurement error and was different from study to study. Besides, it was certainly known that personal pollution exposure levels were very different from those measured at a nearby fixed monitoring station [38]. Thus standardized measurement should be established. (3) In our research, meteorological condition was not included as a covariate, because only some of retrieved studies provided mean or median temperature data. Besides, the association between PM_10_ and stroke attack was different from season to season [16]. Hence, it's not suitable to regard the mean or median temperature data as covariate in our research. Nevertheless, meteorological condition was an important covariate between studies. What's more, age is another important covariate to be considered, but current studies didn't provide sufficient data, they just provide the age range of study objects rather than the mean age. Future researches should provide more accurate data of age, temperature and pay more attention to the influence of age and temperature to stroke attack. (4) We only detect study design and area as important influence factors among PM and stroke by Meta-regression and subgroup analysis, however, there might exist other covariates that we didn't focus on, such as age, historical disease and meteorological condition. Besides, adding more studies may bring us more valuable covariates. (5) Lots of studies showed that different compositions of PM could cause diverse health outcomes. However, till 2012, there were no such articles, except Halonen's [23] study, which could provide detail data concentrating on the short-term effects of some composition of PM on stroke attack. In some studies [10]–[13], [15], [18]–[20], authors just mentioned traffic exhaust emission was the main origin of PM without providing the accurate data about the association between some composition of PM and stroke attack. While in other studies [7], [8], [16], [21], [22], [24]–[26], authors even didn't mention the effect of composition of PM to stroke attack. As a result, we could only focus on the whole PM's effect on stroke attack. We urged future studies should pay more attention to the different composition of PM on health effect.

## Conclusions

This Meta-regression and Meta-analysis raised significant issues that might help guide the future research in this area. PM_2.5_ and PM_10_ exposure had different effect on different stroke subtypes. So it's worthwhile to study the effects of PM to ischemic stroke and hemorrhagic stroke, respectively. Standardizing of exposure measurement, bringing more covariates, exploring mechanisms and adding more studies in respective subtype are needed.

## Supporting Information

File S1 Table S1, sensitivity analysis of Meta-analysis in different particular matter to different stroke types. PM10 indicates particular matter with aerodynamic diameter ≤10 µm; PM2.5, particular matter with aerodynamic diameter ≤2.5 µm; OR, odds ratio; CI, confidence interval. Table S2, meta-regression of different particular matters exposure to different stroke types. PM2.5 indicates particular matter with aerodynamic diameter ≤2.5 µm; PM10, particular matter with aerodynamic diameter ≤10 µm; Coef., regression coefficient; Std. Err., standard error of logOR; CI, confidence interval. *Sex was not contained as a covariate for these studies were all about the whole population.
(DOC)

Checklist S1 Prisma Checklist.
(DOC)
