# Supplementary material for: Short-Term Effects of Particulate Matter on Stroke Attack: Meta-Regression and Meta-Analyses
Source: PLoS One. 2014 May 6;9(5):e95682. doi: 10.1371/journal.pone.0095682 (PMC4011734; doi:10.1371/journal.pone.0095682)
Supplement: File S1 — Table S1, sensitivity analysis of Meta-analysis in different particular matter to different stroke types. PM10 indicates particular matter with aerodynamic diameter ≤10 µm; PM2.5, particular matter with aerodynamic diameter ≤2.5 µm; OR, odds ratio; CI, confidence interval. Table S2, meta-regression of different particular matters exposure to different stroke types. PM2.5 indicates particular matter with aerodynamic diameter ≤2.5 µm; PM10, particular matter with aerodynamic diameter ≤10 µm; Coef., regression coefficient; Std. Err., standard error of logOR; CI, confidence interval. *Sex was not contained as a covariate for these studies were all about the whole population. (DOC) [file pone.0095682.s001.doc]

**Table S1** Sensitivity analysis of Meta-analysis in different particular matter to different stroke types

|  | First Author (published time) | Model | OR (95%CI) | Q | P |
| --- | --- | --- | --- | --- | --- |
| The effect of PM_2.5_ to stroke attack | Total | Random effect model | 0.999 (0.994~1.003) | 67.09 | 0.000 |
|  | Delete Chan (2006)[9] | Random effect model | 0.996 (0.990~1.003) | 58.74 | 0.001 |
|  | Delete Dominici (2006)[17] | Random effect model | 0.998 (0.993~1.003) | 57.54 | 0.003 |
|  | Delete Jalaludin (2006)[18] | Random effect model | 1.003 (0.998~1.007) | 43.09 | 0.026 |
|  | Delete Villeneuve (2006)[19] | Random effect model | 0.998 (0.994~1.003) | 63.92 | 0.000 |
|  | Delete Bell (2008)[20] | Random effect model | 0.999 (0.994~1.005) | 61.96 | 0.000 |
|  | Delete Lisabeth (2008)[22] | Random effect model | 0.998 (0.994~1.003) | 62.09 | 0.001 |
|  | Delete Halonen (2009)[23] | Random effect model | 1.000 (0.995~1.005) | 62.38 | 0.000 |
|  | Delete Villeneuve (2012)[12] | Random effect model | 0.998 (0.994~1.003) | 65.96 | 0.000 |
|  | Delete Wellenius (2012)[13] | Random effect model | 0.998 (0.994~1.003) | 52.40 | 0.007 |
| The effect of PM_10_ to stroke attack | Total | Random effect model | 1.004 (1.000~1.008) | 223.25 | 0.000 |
|  | Delete Wordley (1997)[7] | Random effect model | 1.004 (1.000~1.008) | 220.30 | 0.000 |
|  | Delete Wong (1999)[15] | Random effect model | 1.004 (1.001~1.008) | 223.25 | 0.000 |
|  | Delete Linn (2000)[16] | Random effect model | 1.004 (1.001~1.008) | 222.70 | 0.000 |
|  | Delete Tsai (2003)[8] | Random effect model | 1.002 (0.999~1.005) | 143.77 | 0.000 |
|  | Delete Chan (2006)[9] | Random effect model | 1.004 (1.000~1.008) | 215.71 | 0.000 |
|  | Delete Jalaludin (2006)[18] | Random effect model | 1.006(1.003~1.010) | 197.71 | 0.000 |
|  | Delete Villeneuve (2006)[19] | Random effect model | 1.004 (1.001~1.008) | 218.97 | 0.000 |
|  | Delete Henrotin (2007)[10] | Random effect model | 1.004 (1.001~1.008) | 219.93 | 0.000 |
|  | Delete Bell (2008)[20] | Random effect model | 1.005 (1.001~1.009) | 211.47 | 0.000 |
|  | Delete Guo (2008)[21] | Random effect model | 1.004 (1.000~1.008) | 223.25 | 0.000 |
|  | Delete Ye (2009)[24] | Random effect model | 1.005 (1.001~1.008) | 219.53 | 0.000 |
|  | Delete Andersen (2010)[11] | Random effect model | 1.004 (1.000~1.008) | 210.37 | 0.000 |
|  | Delete Vidale (2010)[25] | Random effect model | 1.002 (0.999~1.006) | 160.42 | 0.000 |
|  | Delete Willocks (2012)[26] | Random effect model | 1.006 (1.001~1.010) | 213.31 | 0.000 |
| The effect of PM_2.5_ to hemorrhagic stroke | Total | Fixed effect model | 0.968 (0.941~0.995) | 13.07 | 0.159 |
|  | Delete Chan (2006)[9] | Fixed effect model | 1.062 (0.993~1.135) | 3.60 | 0.609 |
|  | Delete Villeneuve (2006)[19] | Fixed effect model | 0.958 (0.931~0.987) | 7.18 | 0.305 |
|  | Delete Villeneuve (2012)[12] | Fixed effect model | 0.960 (0.933~0.988) | 7.22 | 0.301 |
| The effect of PM_10_ to hemorrhagic stroke | Total | Random effect model | 1.007 (0.992~1.022) | 39.82 | 0.002 |
|  | Delete Tsai (2003)[8] | Fixed effect model | 0.997 (0.990~1.004) | 11.93 | 0.804 |
|  | Delete Chan (2006)[9] | Random effect model | 1.013 (0.984~1.043) | 36.21 | 0.001 |
|  | Delete Villeneuve (2006)[19] | Random effect model | 1.004 (0.989~1.020) | 36.81 | 0.001 |
|  | Delete Henrotin (2007)[10] | Random effect model | 1.007 (0.991~1.023) | 39.76 | 0.000 |
|  | Delete Ye (2009)[24] | Random effect model | 1.010 (0.994~1.027) | 35.97 | 0.005 |
|  | Delete Andersen (2010)[11] | Random effect model | 1.009 (0.993~1.025) | 33.28 | 0.001 |
| The effect of PM_2.5_ to ischemic stroke | Total | Random effect model | 1.0025 (1.001~1.049) | 24.00 | 0.031 |
|  | Delete Chan (2006)[9] | Fixed effect model | 1.045 (1.017~1.073) | 13.28 | 0.150 |
|  | Delete Villeneuve (2006)[19] | Random effect model | 1.034 (1.004~1.065) | 23.56 | 0.009 |
|  | Delete Lisabeth (2008)[22] | Random effect model | 1.020 (0.994~1.046) | 20.87 | 0.035 |
|  | Delete Villeneuve (2012)[12] | Random effect model | 1.022 (0.995~1.049) | 22.02 | 0.015 |
|  | Delete Wellenius (2012)[13] | Fixed effect model | 1.011 (0.995~1.027) | 11.04 | 0.440 |
| The effect of PM_10_ to ischemic stroke | Total | Random effect model | 1.013 (1.001~1.025) | 98.04 | 0.000 |
|  | Delete Tsai (2003)[8] | Random effect model | 1.011 (1.000~1.022) | 66.48 | 0.000 |
|  | Delete Chan (2006)[9] | Random effect model | 1.016 (1.002~1.030) | 81.16 | 0.000 |
|  | Delete Villeneuve (2006)[19] | Random effect model | 1.016 (1.003~1.029) | 89.35 | 0.000 |
|  | Delete Henrotin (2007)[10] | Random effect model | 1.015 (1.002~1.028) | 93.15 | 0.000 |
|  | Delete Andersen (2010)[11] | Random effect model | 1.013 (0.997~1.029) | 92.95 | 0.000 |
|  | Delete Vidale (2010)[25] | Random effect model | 1.008 (0.996~1.020) | 55.44 | 0.000 |

PM_10_ indicates particular matter with aerodynamic diameter ≤ 10 μm; PM_2.5_, particular matter with aerodynamic diameter ≤ 2.5 μm; OR, odds ratio; CI, confidence interval.

**Table S2** Meta-regression of different particular matters exposure to different stroke types

|  | Covariant | Coef. | Std. Err. | P | (95% CI) |
| --- | --- | --- | --- | --- | --- |
| PM_2.5_ to stroke attack* | lag | -0.001 | 0.002 | 0.743 | (-0.004~0.003) |
|  | area | 0.004 | 0.002 | 0.118 | (-0.001~0.008) |
|  | design | 0.032 | 0.015 | 0.035 | (0.002~0.062) |
|  | research period | 0.022 | 0.021 | 0.295 | (-0.020~0.064) |
| PM_10_ to stroke attack | lag | -0.001 | 0.002 | 0.679 | (-0.004~0.002) |
|  | area | 0.007 | 0.003 | 0.007 | (0.002~0.012) |
|  | sex | -0.011 | 0.014 | 0.404 | (-0.038~0.016) |
|  | design | 0.006 | 0.005 | 0.243 | (-0.004~0.017) |
|  | research period | 0.000 | 0.005 | 0.947 | (-0.010~0.010) |
| PM_10_ to hemorrhagic stroke attack* | lag | -0.011 | 0.006 | 0.061 | (-0.023~0.001) |
|  | area | -0.014 | 0.016 | 0.386 | (-0.047~0.019) |
|  | design | 0.018 | 0.016 | 0.295 | (-0.017~0.053) |
|  | research period | -0.016 | 0.020 | 0.426 | (-0.059~0.026) |
| PM_2.5_ to ischemic stroke attack* | lag | 0.158 | 0.009 | 0.089 | (-0.003~0.034) |
|  | area | -0.044 | 0.021 | 0.055 | (-0.090~0.001) |
|  | design | 0.032 | 0.026 | 0.240 | (-0.024~0.087) |
|  | research period | 0.052 | 0.029 | 0.096 | (-0.011~0.115) |
| PM_10_ to ischemic stroke attack | lag | 0.001 | 0.003 | 0.691 | (-0.005~0.008) |
|  | area | 0.010 | 0.008 | 0.201 | (-0.006~0.026) |
|  | sex | -0.018 | 0.014 | 0.230 | (-0.047~0.019) |
|  | design | -0.007 | 0.011 | 0.519 | (-0.031~0.016) |
|  | research period | 0.003 | 0.011 | 0.767 | (-0.020~0.026) |

PM_2.5_ indicates particular matter with aerodynamic diameter ≤ 2.5 μm; PM_10_, particular matter with aerodynamic diameter ≤ 10 μm; Coef., regression coefficient; Std. Err., standard error of logOR; CI, confidence interval.

*Sex was not contained as a covariate for these studies were all about the whole population.
